# In-hospital and post-discharge outcomes of pediatric acute myocarditis underwent after high-dose steroid or intravenous immunoglobulin therapy

**DOI:** 10.1186/s12872-018-0981-3

**Published:** 2019-01-09

**Authors:** Ming-Shyan Lin, Yu-Hsiang Tseng, Mei-Yen Chen, Chang-Min Chung, Ming-Horng Tsai, Po-Chang Wang, Jung-Jung Chang, Tien-Hsing Chen, Yu-Sheng Lin

**Affiliations:** 1 Department of Cardiology, Chiayi Chang Gung Memorial Hospital, No. 6, Sec. West, Chai-Pu Road, Pu-TZ City, Chai Yi Hsien, Taiwan; 2 Department of Nursing, Chang Gung University of Science and Technology, Taoyuan, Taiwan; 3 Department of Nursing, Chang Gung University, Taoyuan, Taiwan; 4 Department of Pediatrics, Chang Gung Memorial Hospital, Yunlin,, Taiwan; 5 Department of Cardiology, Chang Gung Memorial Hospital, Keelung, Taiwan

**Keywords:** Acute myocarditis, Immunoglobulin, Immunotherapy, Immunosuppression, Propensity score analysis

## Abstract

**Background:**

High-dose steroids and intravenous immunoglobulin (IVIG) are controversial treatments for pediatric patients with acute myocarditis. This study aimed to investigate their efficacies in the Taiwanese pediatric population.

**Methods:**

This study evaluated 5563 acute myocarditis patients from the Taiwan’s National Health Insurance Research Database and identified 1542 pediatric patients hospitalized for acute myocarditis between January 1, 2001 and December 31, 2011. The exclusion criteria were age of > 11 years, associated cardiovascular comorbidities, autoimmune disease, malignancy before the index hospitalization, extracorporeal membrane oxygenation, intra-aortic balloon pumping, and dual therapy using IVIG and high-dose steroids.

**Results:**

After 2:1 propensity score matching, we identified 208 subjects without steroid therapy and 104 subjects who received high-dose steroids. The mean age in that cohort was 2.6 ± 2.9 years, and high-dose steroid therapy had no significant effects on major in-hospital complications and post-discharge outcomes. After 2:1 propensity score matching, we identified 178 subjects without IVIG therapy and 89 subjects who received IVIG. The mean age in that cohort was 2.0 ± 2.1 years, and IVIG had no significant effects on the major outcomes.

**Conclusions:**

The present study revealed that high-dose steroid or IVIG therapy had no significant effects on major in-hospital complications, late heart failure hospitalization, and long-term mortality.

## Background

Acute myocarditis is defined as inflammation of the myocardium that is related to viral infection and autoimmune reactions [1], which may lead to the sudden onset of heart failure, arrhythmia, fulminant hemodynamic collapse, and death. This condition is common in the pediatric population, as approximately 55% of autopsied pediatric patients were diagnosed with myocarditis [2]. Although most children can recover from acute myocarditis after treatment, approximately 9% of patients will progress to dilated cardiomyopathy during mid-term and long-term follow-up. Therefore, it is important to manage the complications of acute myocarditis in this population. The current recommendations for pediatric myocarditis emphasize primary treatment using supportive medical therapy [3], which can involve inotropic agents for acute heart failure and mechanical circulatory support (MCS) for severe cases.

Immunotherapies are occasionally used in cases with residual refractory and chronic cardiac dysfunction. For example, high-dose steroids reportedly have a therapeutic effect in pediatric myocarditis cases [4–7], and can promote the recovery of left ventricle systolic function in acute myocarditis [8]. However, recent reviews and meta-analyses have indicated that high-dose steroid therapy did not significantly influence clinical outcomes [9] and did not reduce the mortality rate [10, 11]. Nevertheless, despite the controversial results among pediatric patients with acute myocarditis, high-dose steroids are used in 25–30% of American pediatric myocarditis cases [12]. Intravenous immunoglobulin (IVIG) is another immune-modulating therapy that may be used for pediatric acute myocarditis based on its anti-viral and anti-inflammatory effects [13]. However, a recent study failed to reveal a survival benefit associated with IVIG therapy for pediatric myocarditis [14]. Despite these heterogeneous results, all of the related studies regarding IVIG or high-dose steroid therapy have been limited by their small size and matched-controlled designs. Therefore, the present study aimed to evaluate the effects of high-dose steroid and IVIG therapy on Taiwanese pediatric patients with acute myocarditis using data from a nationally representative cohort.

## Material and methods

### Data source

This nationally representative population-based retrospective cohort study evaluated data retrieved from the Taiwan’s National Health Insurance Research Database (NHIRD), which is maintained by the Taiwan National Health Research Institute. The NHIRD contains healthcare information for 99.91% of the Taiwanese population who are enrolled in the National Health Insurance (NHI) program (approximately 23.20 million people in 2012). The NHIRD records all medical data, including records for inpatient and outpatient care, and basic data regarding age, sex, underlying diseases, and the treating hospital’s characteristics. The accuracy and validity of the NHIRD data is regularly evaluated by the NHI Bureau [15]. The present study evaluated all patients who were admitted to hospital for treatment of acute myocarditis. The study’s retrospective protocol was approved by the institutional review board of Chang Gung Memorial Hospital (103-6244B) and the need for written informed consent was waived by the ethics committee due to all NHIRD data had been de-identified for research purposes.

### Study population and definitions

**Fig. 1:**
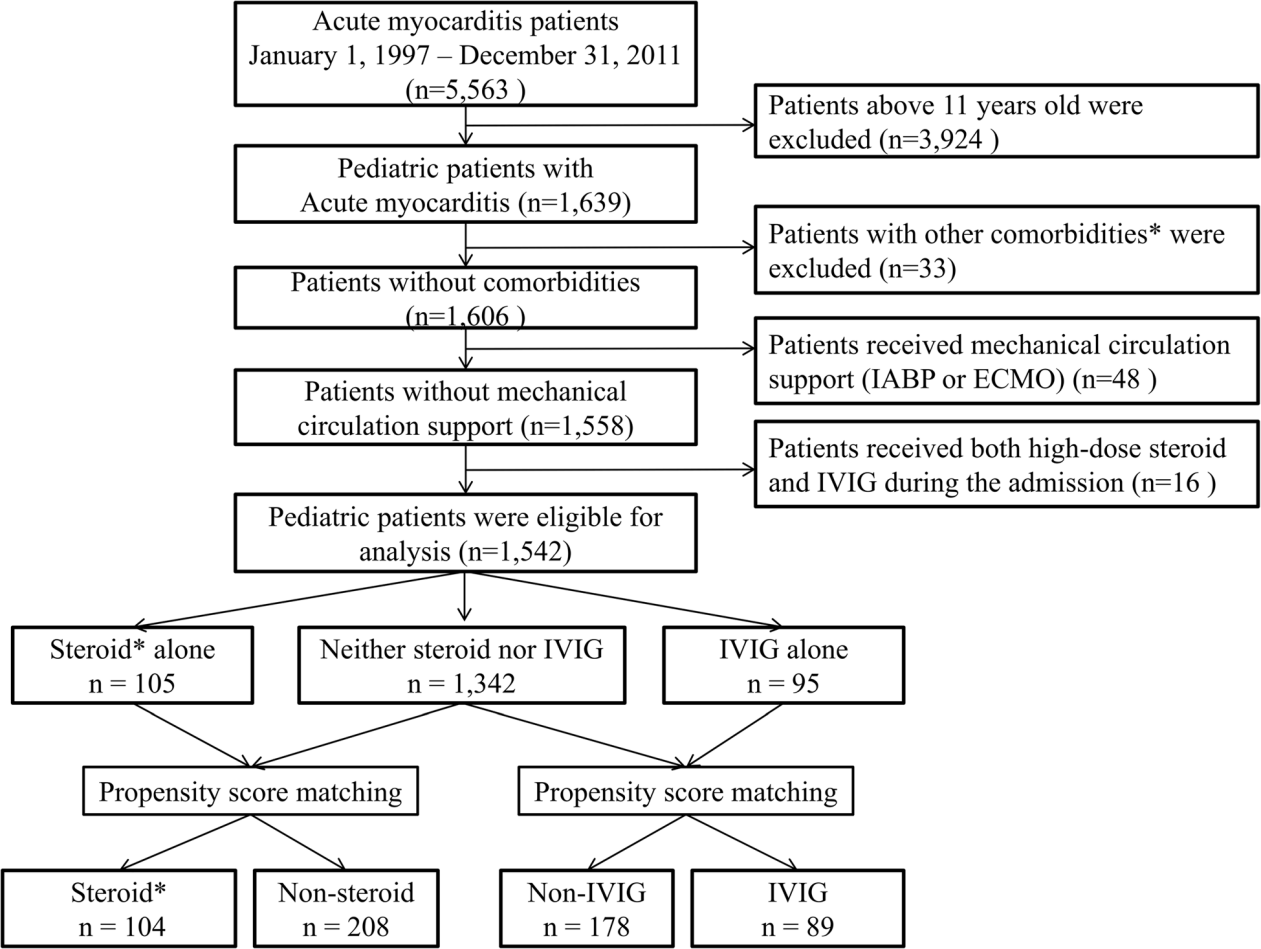
Flow chart of the study design and enrollment. *Comorbidities: Diabetes mellitus, hypertension, hyperlipidemia, ischemic heart disease, heart failure, cerebrovascular accident, renal failure, autoimmune disease, malignancy. *Steroid: High-dose steroid. ECMO:extra-corporeal membrane oxygenation; IVIG:intravenous immunoglobulin; IABP:intraaortic balloon pumping

Between January 1, 2001 and December 31, 2011, 5563 patients were hospitalized for acute myocarditis and were identified using the International Classification of Diseases, Ninth Revision code (422.0), which was validated in a previous study [16]. The exclusion criteria at the index hospitalization were age of > 11 years, diabetes mellitus (250), hypertension (401–405), hyperlipidemia (272.0–272.2), ischemic heart disease (410–414), heart failure (428), cerebrovascular accident (430–438), renal failure (584–586), autoimmune disease (136.1, 443.1, 446.0, 446.2, 446.4, 446.5, 446.7, 555, 556, 694.4, 710.0, 710.1, 710.2, 710.3, 710.4, 714.0), malignancy (140–208), extracorporeal membrane oxygenation, intra-aortic balloon pumping, and a combination of IVIG and high-dose steroid therapy. The remaining patients were grouped according to whether they received no high-dose steroids or IVIG (no immunotherapy), only high-dose steroid treatment (steroids alone), or only IVIG (IVIG alone). High-dose steroid therapy was defined as intravenous prednisolone used at > 10 mg/kg/day according to the patient’s average body weight. The use of IVIG was defined as IVIG treatment during the index hospitalization. The study flow chart is shown in Fig. 1. All clinical comorbidities, complications, and outcomes were identified using the International Classification of Diseases, Ninth Revision codes, and steps for managing acute myocarditis during the admission were recorded as completely as possible. These steps included confirmatory examinations (virus detection, cardiac magnetic resonance imaging, and cardiac biopsy), medication-based treatments (any intravenous inotropic agents, high-dose steroids, and IVIG), use of a temporary pacing device, and resuscitation methods (electrical cardioversion, intubation with ventilation support, and cardiopulmonary resuscitation [CPR]).

### Study design

Two cohorts were evaluated in this study. The first cohort was used to assess the effects of high-dose steroid therapy on pediatric acute myocarditis, while the second cohort was used to assess the effects of IVIG. To reduce potential confounding and selection biases, because the two groups were not randomized, propensity scoring matching (PSM) was performed [17]. Each patient in the high-dose steroid or IVIG groups was matched to 2 control patients who did not receive high-dose steroids or IVIG (i.e., no immunotherapy) using the probability calculated via logistic regression based on the following confounding variables: sex, age, anti-heart failure medications (ACEi/ARB, beta blocker, digoxin, loop diuretics, and potassium-sparing diuretics), inotropic agents (dobutamine, milrinone, dopamine, norepinephrine), severity assessment (ventricular tachycardia/fibrillation [VT/VF], CPR, bradycardia, cardioversion, temporary pacemaker, and intensive care unit [ICU] admission), and the year of the index hospitalization. The matching procedure was performed using SAS (version 9.3; SAS Institute, Cary, NC).

### Outcome assessments

The outcomes of interest were defined as in-hospital complications and discharge outcomes. In-hospital complications were defined as new-onset acute renal failure, new onset dialysis, stroke, and cardiovascular death. Discharge outcomes were defined as any clinical event after the index hospitalization, such as high-grade AV block, VT/VF, use of dialysis, heart failure hospitalization, stroke, heart transplantation, cardiovascular death, and all-cause mortality. Cardiovascular death was defined as death resulting from acute myocardial infarction, sudden cardiac death, heart failure, pulmonary embolism, dysrhythmia unrelated to sudden cardiac death, stroke, and peripheral artery disease. All-cause mortality was defined as death from any cause within 30 days after the index admission date, regardless of whether the patient died during the hospitalization or after discharge.

### Statistical analysis

The pre-matching clinical characteristics were compared between the immunotherapy and non-immunotherapy groups using Fisher’s exact test for categorical variables and using one-way analysis of variance for continuous variables. The clinical characteristics were compared between the immunotherapy groups (high-dose steroids and IVIG) and the matched controls using Fisher’s exact test for categorical variables and using the t-test for continuous variables. The in-hospital outcomes were compared between the study groups using multivariable logistic regression analysis. The continuous in-hospital outcomes (i.e., days of ventilator support) were compared between the study groups using multivariable linear regression analysis. Multivariable Cox proportional hazard models were used to compare the times to the first occurrence of the various outcomes between the study groups. All multivariable analyses were adjusted for the subjects’ propensity scores [17]. The PSM was performed based on the nearest-neighbor method (greedy matching) and a caliper radius of 0.5 sigma, which signifies the maximum tolerated distance in the propensity score. All data analyses were performed using IBM SPSS software (version 22; IBM Corp., Armonk, NY).

## Results

The present study evaluated 1542 pediatric patients who were hospitalized for acute myocarditis between January 1, 2001 and December 31, 2011. The baseline characteristics are listed in Table 1. Patients who received immunotherapy were younger than the patients who did not receive immunotherapy (high-dose steroids alone: 2.6 ± 2.9 years, IVIG alone: 1.9 ± 2.1 years, no immunotherapy: 2.9 ± 2.6 years).

**Table 1:** Demographic and clinical data of the study patients before propensity score matching

Variable	Neither steroid nor IVIG	Steroid alone*	IVIG alone	*P*‡
Number of patients	1342	105	95	–
Male gender—no. (%)	713 (53.1)	57 (54.3)	62 (65.3)	0.072
Age (year)	2.9 ± 2.6	2.6 ± 2.9	1.9 ± 2.1	< 0.001
Age group—no. (%)				< 0.001
< 1 yrs.	394 (29.4)	44 (41.9)	39 (41.1)	
1~2 yrs.	432 (32.2)	33 (31.4)	42 (44.2)	
3~5 yrs.	337 (25.1)	12 (11.4)	7 (7.4)	
6~11 yrs.	179 (13.3)	16 (15.2)	7 (7.4)	
Oral Medication—no. (%)				
ACEi/ ARB	66 (4.9)	10 (9.5)	5 (5.3)	0.131
Beta blocker	58 (4.3)	6 (5.7)	4 (4.2)	0.723
Digoxin	66 (4.9)	14 (13.3)	6 (6.3)	0.003
Loop diuretics	50 (3.7)	15 (14.3)	6 (6.3)	< 0.001
Potassium-sparing diuretics	9 (0.7)	4 (3.8)	1 (1.1)	0.017
Intravenous agents—no. (%)				
Dobutamine	68 (5.1)	32 (30.5)	13 (13.7)	< 0.001
Milrinone	31 (2.3)	7 (6.7)	11 (11.6)	< 0.001
Dopamine	127 (9.5)	40 (38.1)	23 (24.2)	< 0.001
Norepinephrine	4 (0.3)	2 (1.9)	0 (0.0)	0.104
Examination—no. (%)				
Cardiac MRI	18 (1.3)	5 (4.8)	9 (9.5)	< 0.001
Cardiac biopsy	1 (0.1)	1 (1.0)	0 (0.0)	0.243
Virus detection	404 (30.1)	53 (50.5)	40 (42.1)	< 0.001
Severity assessment—no. (%)				
VT/VF	9 (0.7)	1 (1.0)	1 (1.1)	0.428
CPR	38 (2.8)	12 (11.4)	6 (6.3)	< 0.001
Bradycardia	13 (1.0)	2 (1.9)	1 (1.1)	0.489
Cardioversion	20 (1.5)	6 (5.7)	4 (4.2)	0.005
Temporary Pacemaker	11 (0.8)	2 (1.9)	0 (0.0)	0.375
ICU stay	304 (22.7)	65 (61.9)	28 (29.5)	< 0.001

‡ Fisher’s exact test for categorical variable or one-way ANOVA for continuous variable;

*Steroid alone: those patients received high-dose steroid therapy

Abbreviations: *ACEi* angiotensin-converting enzyme inhibitor), *ARB* angiotensin receptor blocker, *CPR* cardiopulmonary resuscitation, *ICU* intensive care unit, *IVIG* intravenous immunoglobulin, *MRI* magnetic resonance imaging, *VT/VF* ventricular tachycardia/ventricular fibrillation

Patients who only received high-dose steroids were more likely to receive inotropic agents (dobutamine or dopamine) than patients who received IVIG or no immunotherapy. However, patients who received high-dose steroids were also more likely to be admitted to the ICU than patients who received IVIG (61.9% vs 29.5%). Relative to patients who received no immunotherapy, patients who received immunotherapy had a similar incidence of cardiac arrhythmia (VT, VF, and bradycardia) but were more likely to require resuscitation (cardiac massage and cardioversion) (*P* < 0.005). Less than 10% of the subjects received standard medications for heart failure (ACEi/ARB, beta blockers, potassium-sparing diuretics), and digoxin plus loop diuretics were common medications. A small number of patients underwent cardiac magnetic resonance imaging and cardiac biopsy.

### Effects of high-dose steroids in pediatric patients with acute myocarditis

**Fig. 2:**
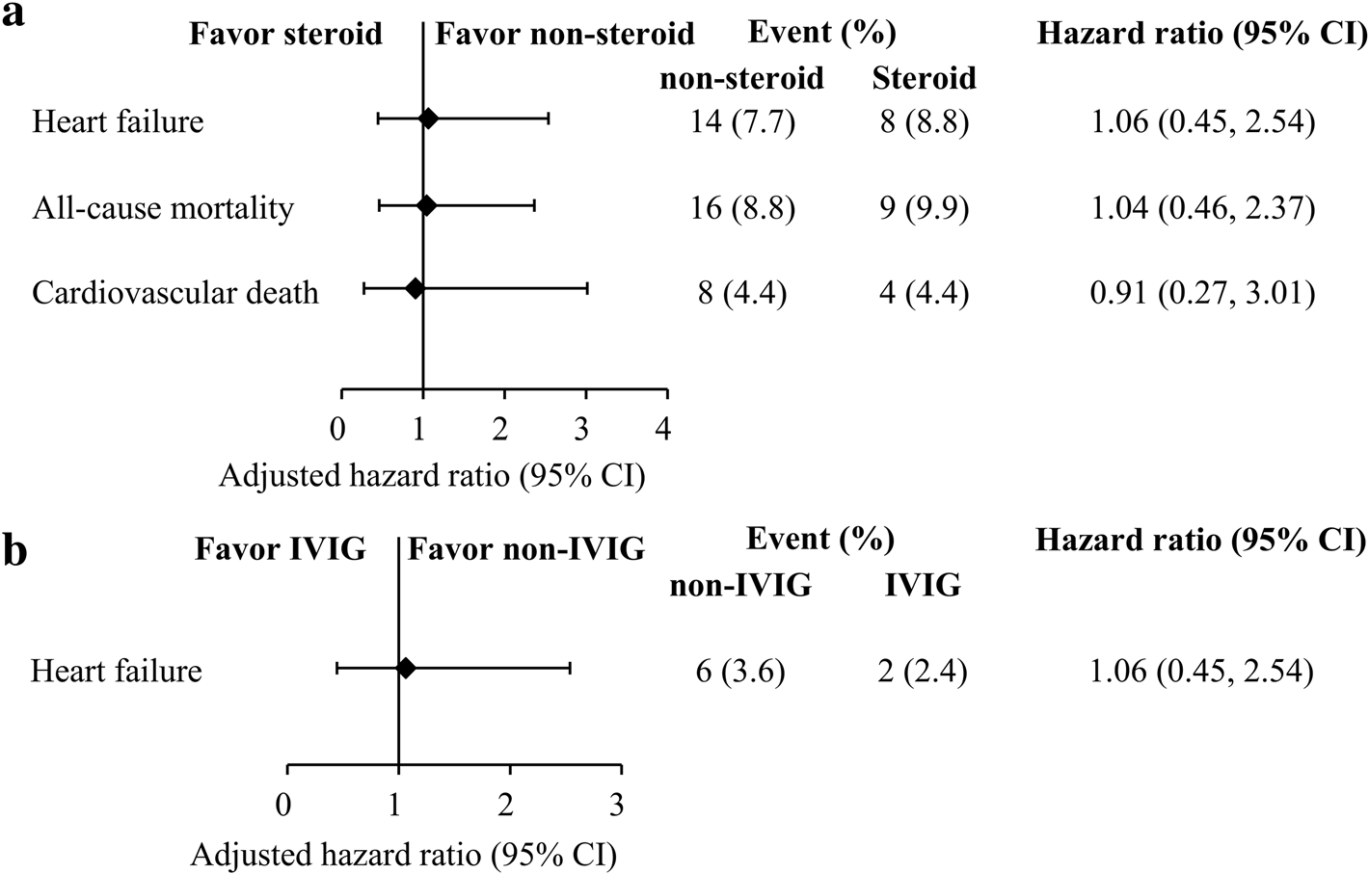
Multivariable stratified analyses for the association between immunotherapies and discharge outcomes. (**a**) Steroid therapy versus non-steroid therapy and (**b**) IVIG therapy versus non-IVIG. Some variables did not showed here due to hazard ratio cannot be calculated based on no events. *Steroid: High-dose steroid

The PSM process matched 104 patients who received high-dose steroids to 208 patients who received no immunotherapy (Fig. 1). Table 2 shows that there were no significant differences between the groups in terms of their baseline characteristics, age, anti-heart failure medications, inotropic agent use, and severity measures. The mean age was 2.6 ± 3.0 years and > 70% of the patients were toddlers (< 2 years old). Table 3 shows that there were no significant differences in the two groups’ in-hospital complications, including acute renal failure, new onset dialysis, and heart transplantation. Furthermore, there was no significant difference in the in-hospital mortality rates (*P* = 0.758). Post-discharge VT/VF, high-degree AV block, and renal failure requiring dialysis were rare. There were also no significant differences in the incidences of heart failure hospitalization, cardiovascular death, and all-cause mortality (Fig. 2a, Table 4).

**Table 2:** Demographic and clinical data of the study patients after propensity score matching

	First cohort			Second cohort		
Variable	Non-Steroid*	Steroid*	*P*‡	Non-IVIG	IVIG	*P*‡
Number of patients	208	104	–	178	89	–
Male gender—no. (%)	108 (51.9)	56 (53.8)	0.810	111 (62.4)	58 (65.2)	0.688
Age (year)	2.4 ± 2.8	2.6 ± 2.9	0.660	1.7 ± 1.9	2.0 ± 2.1	0.313
Age group			0.875			0.576
< 1 yrs.	87 (41.8)	44 (42.3)		79 (44.4)	35 (39.3)	
1~2 yrs.	67 (32.2)	32 (30.8)		80 (44.9)	40 (44.9)	
3~5 yrs.	28 (13.5)	12 (11.5)		8 (4.5)	7 (7.9)	
6~11 yrs.	26 (12.5)	16 (15.4)		11 (6.2)	7 (7.9)	
Oral Medication—no. (%)						
ACEi/ ARB	22 (10.6)	10 (9.6)	0.846	8 (4.5)	5 (5.6)	0.765
Beta blocker	8 (3.8)	6 (5.8)	0.563	7 (3.9)	4 (4.5)	1.000
Digoxin	29 (13.9)	14 (13.5)	1.000	12 (6.7)	5 (5.6)	0.797
Loop diuretics	26 (12.5)	15 (14.4)	0.723	9 (5.1)	6 (6.7)	0.581
Potassium-sparing diuretics	6 (2.9)	4 (3.8)	0.736	2 (1.1)	1 (1.1)	1.000
Intravenous agents—no. (%)						
Dobutamine	56 (26.9)	31 (29.8)	0.595	18 (10.1)	10 (11.2)	0.833
Milrinone	13 (6.3)	7 (6.7)	1.000	7 (3.9)	6 (6.7)	0.369
Dopamine	77 (37.0)	39 (37.5)	1.000	29 (16.3)	17 (19.1)	0.607
Norepinephrine	3 (1.4)	2 (1.9)	1.000	0 (0.0)	0 (0.0)	–
Examination—no. (%)						
Cardiac MRI	6 (2.9)	5 (4.8)	0.516	3 (1.7)	7 (7.9)	0.018
Cardiac biopsy	0 (0.0)	1 (1.0)	0.333	0 (0.0)	0 (0.0)	–
Virus detection	107 (51.4)	52 (50.0)	0.812	54 (30.3)	34 (38.2)	0.215
Severity assessment—no. (%)						
VT/VF	5 (2.4)	1 (1.0)	0.668	3 (1.7)	1 (1.1)	1.000
CPR	25 (12.0)	11 (10.6)	0.851	9 (5.1)	5 (5.6)	1.000
Bradycardia	6 (2.9)	2 (1.9)	0.723	1 (0.6)	1 (1.1)	1.000
Cardioversion	16 (7.7)	6 (5.8)	0.643	5 (2.8)	3 (3.4)	1.000
Temporary pacemaker	6 (2.9)	2 (1.9)	0.723	1 (0.6)	0 (0.0)	1.000
ICU stay	130 (62.5)	64 (61.5)	0.902	42 (23.6)	23 (25.8)	0.762

‡ Fisher’s exact test for categorical variable or t-test for continuous variable;

*Steroid: patients received high-dose steroid therapy; non-steroid: patients without receiving high-dose steroid therapy

Abbreviations: *ACEi* angiotensin-converting-enzyme inhibitor), *ARB* angiotensin receptor blocker, *CPR* cardiopulmonary resuscitation, *ICU* intensive care unit, *IVIG* intravenous immunoglobulin, *MRI* magnetic resonance imaging, *VT/VF* ventricular tachycardia/ventricular fibrillation

**Table 3:** In hospital outcomes

	First cohort study			Second cohort study		
Variable	Non-Steroid*	Steroid*	*P*‡	Non IVIG	IVIG	*P*‡
Number of patients	208	104	–	178	89	–
Acute renal failure —no. (%)	1 (0.5)	1 (1.0)	0.624	0 (0.0)	0 (0.0)	–
New onset of dialysis —no. (%)	0 (0.0)	0 (0.0)	–	0 (0.0)	0 (0.0)	–
Heart transplantation—no. (%)	0 (0.0)	0 (0.0)	–	0 (0.0)	0 (0.0)	–
In hospital death—no. (%)	27 (13.0)	13 (12.5)	0.758	10 (5.6)	5 (5.6)	0.758

‡ Adjusted for propensity score (logistic regression for binary outcome or linear regression for continuous outcome);

*Steroid: patients received high-dose steroid therapy; non-steroid: patients without receiving high-dose steroid therapy

Abbreviations: *IVIG* intravenous immunoglobulin

**Table 4:** Discharge outcomes for those survived during the index admission

	First cohort			Second cohort		
Variable	Non-Steroid*	Steroid*	*P*‡	Non-IVIG	IVIG	*P*‡
Number of patients	181	91	–	168	84	–
VT/VF—no. (%)	2 (1.1)	0 (0.0)	–	0 (0.0)	0 (0.0)	–
High-grade AVB—no. (%)	1 (0.6)	0 (0.0)	–	0 (0.0)	0 (0.0)	–
Cardiac arrest—no. (%)	0 (0.0)	1 (1.1)	–	0 (0.0)	0 (0.0)	–
Heart failure—no. (%)	14 (7.7)	8 (8.8)	0.891	6 (3.6)	2 (2.4)	0.581
Heart transplantation—no. (%)	0 (0.0)	0 (0.0)	–	0 (0.0)	0 (0.0)	–
Any dialysis—no. (%)	0 (0.0)	0 (0.0)	–	0 (0.0)	0 (0.0)	–
New occurrence of dialysis	0 (0.0)	0 (0.0)	–	0 (0.0)	0 (0.0)	–
All-cause mortality	16 (8.8)	9 (9.9)	0.919	8 (4.8)	0 (0.0)	–
CV death	8 (4.4)	4 (4.4)	0.871	4 (2.4)	0 (0.0)	–

‡ Adjusted for propensity score (Cox regression);

*Steroid: those patients received high-dose steroid therapy; non-steroid: those patients without receiving high-dose steroid therapy

Abbreviations: *AVB* atrioventricular block, *CV* cardiovascular, *IVIG* intravenous immunoglobulin, *VT* ventricular tachycardia, *VF* ventricular fibrillation

### Effects of IVIG in pediatric patients with acute myocarditis

The PSM process matched 89 patients who received IVIG alone to 178 patients who received no immunotherapy. The two groups were well balanced in terms of the patients’ clinical characteristics (Table 2). Most patients were male (> 60%) and the mean age was 2.0 ± 2.1 years (> 70% of patients were < 2 years old). The two groups did not include any patients who developed acute renal failure, new onset dialysis, or heart transplantation, and both groups had the same rate of in-hospital mortality (5.6%) (Table 3). The two groups also did not have any patients who developed post-discharge VT/VF, high-grade AV block, and renal failure requiring dialysis, and there was no difference in the incidences of heart failure hospitalization (Fig. 2b). Table 4 shows that the IVIG alone group did not experience any deaths (0/84), although 8 patients in the control group died, including 4 cases (2.4%) of cardiovascular death.

## Discussion

This well-matched nationally representative retrospective cohort study revealed that both immunotherapies (high-dose steroids and IVIG) might not affect the real-world rates of in-hospital mortality and post-discharge hospitalization for late heart failure. Nevertheless, despite the neutral effect of high-dose steroids on long-term all-cause mortality and cardiovascular death, the IVIG group appeared to experience fewer deaths than the PSM control group.

### Study population and design

The present study aimed to evaluate the effects of IVIG and high-dose steroids, which prompted us to aim to exclude potentially confounding baseline characteristics. Aside from baseline characteristics, the usage and duration of MCS were considered the most important confounding factors. This is because MCS is a main therapy for severe cardiogenic shock in pediatric acute myocarditis, and is known to influence the rates of in-hospital mortality and long-term survival [18]. However, the duration of MCS was difficult to account for based on its relative rarity, which led us to exclude patients who received MCS (i.e., extracorporeal membrane oxygenation and intra-aortic balloon pumping). Previous studies regarding acute myocarditis in the pediatric population have also been limited by their small sample size and the emergent nature of acute myocarditis. Therefore, we used PSM to reduce any selection bias in this cohort study.

Endomyocardial biopsy and viral detection are usually recommended for acute myocarditis, although patients in this study rarely underwent cardiac biopsy. Despite endomyocardial biopsy being considered safe in the adult population [19], its rarity for diagnosing myocarditis in pediatric patients may be related to the high risk of myocardial injury that is associated with the lack of available facilities and clinical experience [20]. Furthermore, although many patients underwent testing to detect viral infection, which was typically performed within 3 days in clinical practice, we could not obtain the related laboratory data. Therefore, the biopsy and viral detection statuses were included in the PSM to eliminate their influences on clinical outcomes.

Moreover, the present study included only a small proportion of patients who received medication for heart failure. This is likely related to the high spontaneous recovery rate after discharge, drug intolerance in very young patients, and a lack of randomized controlled trials in the pediatric population. Nevertheless, the findings of our study reflect the real-world practice in Taiwan.

### Outcomes related to high-dose steroid therapy

The pathology of myocarditis can be divided into three phases that involve the host’s innate immune response to virus-infected cells and the subsequent autoimmune reactions [21]. Because immune dysfunction may play a primary role in many cases of myocarditis, immunotherapy may shorten its course, lessen its severity, and improve long-term outcomes. In this context, prednisolone mimics natural steroids during the response to stress and help reduce the subsequent host inflammatory response, even though there are several well-known adverse events. Gagliardi et al. have reported that immunosuppression therapy improved left ventricular function and survival in 68–100% of children [22]. Burch et al. have also suggested that immunosuppression is safe in pediatric subjects with acute myocarditis, although the therapeutic effects were unclear [23]. Unfortunately, a recent systemic review and meta-analysis revealed that immunosuppressive treatment might help improve left ventricular systolic function and clinical symptoms, but did not significantly influence mortality and the incidence of heart transplantation in 3 studies [10, 11]. However, many studies were limited by their small sample size, lack of control groups, short follow-up duration, heterogeneous outcome definitions, and variability in the therapeutic regimens. The present study addressed the issues of small sample sizes and lack of comparable control groups, which revealed that use or non-use of high-dose steroid treatment did not significantly influence the rates of in-hospital mortality, subsequent heart failure hospitalization, or all-cause mortality.

### Outcomes related to IVIG

Because IVIG has anti-inflammatory, immune-modulating, and anti-oxidative stress effects, it seems reasonable that it would be helpful during the initial phase of myocarditis in terms of improving left ventricle function [24–26, 31]. Other studies have demonstrated that IVIG could improve cardiovascular outcomes by decreasing the risk of cardiogenic shock, fatal arrhythmia, or complete heart block [27–30]. However, Yu et al. [28] detected no significant difference in the mortality rates of groups that did and did not receive IVIG (2/32 vs 7/26; *P* = 0.072). Another recent multi-center study also revealed no significant survival benefit from IVIG treatment for pediatric patients with acute myocarditis [31]. However, these studies were also limited by their small sample sizes and the lack of control groups. The present study addressed those limitations using PSM in a nationally representative cohort, which revealed that IVIG did not significantly influence the rates of in-hospital mortality and post-discharge late heart failure hospitalization. Nevertheless, given the absence of cases with late mortality in the IVIG group, further large prospective studies are needed to evaluate its potential survival benefit.

### Study limitations

This study has several limitations. First, the data were retrospectively retrieved from a large administrative database that depends on the coding accuracy of the participating hospitals, and the diagnoses are not as clearly validated as those in prospective registries or studies. However, medical payment directed from medical managements of individual diagnosis is strictly controlled by Taiwan National Health Insurance’s provision. Therefore, though we can’t determine whether the diagnosis was made according to the European Society of Cardiology (ESC) take force proposed criteria in our insurance database [32], we assumed that the wrong diagnosis from clear criteria to be less likely. In addition, pediatric subjects have fewer comorbidities than adult patients, and patients with complex pre-admission comorbidities were excluded to eliminate any related issues. Second, the NHIRD does not include detailed information regarding hemodynamic status, laboratory data (including cardiac troponin levels), clinical severity assessments, treatment, or response. Although high cardiac troponin levels are reportedly associated with poor cardiac function in acute phase, several other studies have indicated that troponin levels did not influence long-term clinical outcomes [33–36]. Nevertheless, the PSM was adjusted for several variables that are reportedly related to outcomes (age distribution, anti-heart failure medication, and usage of inotropic agents) and severity (VT/VF, bradyarrhythmia, CPR use, and ICU stay). Third, we could not assess echocardiographic results or other images to predict prognosis, although a large study has demonstrated that those parameters (e.g., left ventricular ejection fraction, end-diastolic pressure, and end-diastolic dimension index) were not related to poor outcomes [37]. Finally, we did not have access to data regarding biopsy and viral testing results, which precluded any confirmation of the response to immunosuppressive therapy (e.g., development of giant myocarditis, eosinophilic myocarditis, or autoimmune related myocarditis). Thus, to address this issue, we excluded patients with autoimmune diseases before or after the index admission. Furthermore, the sensitivity of endocardial biopsy is limited under the Dallas criteria or for viral detection.

## Conclusion

The lack of conclusive data has led to controversy regarding the efficacy of immunomodulatory therapy (e.g., IVIG or high-dose steroids) for pediatric myocarditis. This Taiwanese– retrospective cohort study provides real-world clinical evidence that high-dose steroid treatment or IVIG treatment have neutral effects on the rates of in-hospital complications and post-discharge outcomes among pediatric patients with acute myocarditis. These results may help guide the approach to using these treatments in clinical practice.

## Abbreviations

aOR: Adjust odds ratio
AV block: Atrioventricular block
CPR: Cardiac pulmonary resuscitation
ECMO: Extra-corporeal membrane oxygenation
IABP: Intraaortic balloon pumping
ICD: International classification of diseases
IVIG: Intravenous immunoglobulin
MCS: Mechanical circulation support
NHI: National Health Institute
NHIRD: National Health Insurance Research Database
PSM: Propensity scoring method
VT/VF: Ventricular tachycardia/fibrillation
